# Proceedings of the Society of Dental Surgeons of the State of New York

**Published:** 1848-10

**Authors:** J. G. Ambler

**Affiliations:** Secretary.


### ARTICLE VIII.

### *Proceedings of the Society of Dental Surgeons of the State of New York.*

At the annual meeting of this Society, held at the rooms of the College of Pharmacy, No. 411 Broadway, on Tuesday, September 12th, the Society was called to order by the President.

The Report of the Treasurer being called for, was read and accepted.

| | |
|---|---|
| [Receipts from every source, . . . . | $ 320 00 |
| Disbursements, . . . . . . | 18 62 |
| Amount remaining in the Treasury, . . . | $ 301 38 |
| Amount due the Society for initiation fees and yearly dues, . . . . . . . | 32 00] |

The Committee on Mineral Teeth reported progress, and was continued.

A communication from W. A. Kentish, relating to Dr. Levett's enamelled plates, was referred to the committee upon that subject, appointed at a former meeting.

The President, (Dr. Covill,) then addressed the Society, in an eloquent and forcible manner, upon the advantages of association among dental surgeons, and the necessity of strict professional integrity.

The Executive Committee then presented their report, which was accepted.

[The report of the Executive Committee contained the following suggestions and resolutions for the consideration of the Society:

1st. Your Committee suggests that the Executive Committee be authorized to procure a suitable room, and have it properly arranged and furnished for a Dental Lyceum, to contain a Library, Museum, and conveniences for practical operations in Dental Surgery, to be performed before any members of the Society who may choose to be present.

2d. In order that the Museum may be interesting and instructive, members of the profession be invited to present to the Society any specimens of disease or deformity, either of teeth, maxillary bones, or other parts, which they may be willing to part with; also such instrument, whether ancient or modern, as will be calculated to give an idea of the state of the profession in the various periods of its rise and progress.

*Resolved*, That the Executive Committee be requested to make arrangements with some of the benevolent institutions for furnishing subjects for practical operations before the Society.

*Resolved*, That every member of the Society who will devote, at the rooms of the Society, one or more days in a year, shall receive the thanks of the Society.

*Resolved*, That the Society charge for its public operations, the actual cost of materials, and no more.

*Resolved*, That the operations performed at the rooms of the Society, be open to the inspection of all its members.]

A letter was read from Dr. S. Mapes, regretting his inability to attend the meeting of the Society.

Dr. F. H. Clark then read an Essay on Mechanical Dentistry.

Dr. J. Lovejoy then read his Essay on the Importance of Filling Teeth.

The Society then, in committee of the whole, took up the suggestions contained in the report of the Executive Committee, and after some time spent in discussing them, the following resolutions were reported and adopted by the Society:

1st. *Resolved*, That the Executive Committee be authorised to rent a room, at an annual expense not exceeding one hundred and twenty-five dollars, for the permanent use of the Society, and that they be further empowered to furnish it with seats and other necessary articles, at an expense not exceeding fifty dollars.

2d. *Resolved*, That the suggestions relative to contributions to the Dental Museum be adopted.

3d. *Resolved*, That the Executive Committee be requested to ascertain whether arrangements can be made with some of the benevolent institutions, for furnishing patients for clinical operations, to be performed before the Society.

On motion, *Resolved*, That when this Society adjourn, it do so to meet at 7 o'clock this evening.

Dr. Allen submitted the following resolution, to be acted upon, (according to the requirements of the Constitution,) at the next annual meeting.

*Resolved*, That the twelfth article of the Constitution of this Society be amended by omitting the word "annual" in the second line, and substituting the word *regular* for the word "annual," in the third line. Dr. Clark also submitted the following:

*Resolved*, That the ninth article of the Constitution of this Society be amended, by adding after the word "regulation"—It shall have power to grant diplomas or certificates of membership, under such restrictions as the Society shall impose.

Dr. Clark offered the following, to be acted upon at the next regular meeting:

*Resolved*, That the sum of one hundred dollars be appropriated out of the funds of the Society, for the improvement of mineral teeth.

On motion adjourned to 7 o'clock.

At 7 o'clock the Society was called to order by the President.

The Librarian, as chairman of the Library Committee, presented his report, which was accepted.

[The Librarian, through the hands of Dr. Hawes, has received about one hundred volumes, (including pamphlets,) on the subject of surgical and mechanical dentistry, most of which, with the names of the donors, have been acknowledged through the Dental Recorder.

There has also been subscribed in cash, the amount of fifty-six dollars, nineteen of which have been paid, viz. Mr. A. Jones, $10; Mr. J. Alcock, $5; Dr. Covill, $2; Dr. E. Baker, $2.

The balance will be acknowledged when received, as also several more books. The Committee are about arranging and numbering the books, so that in a few weeks they will be ready for delivery to the members.]

The annual address was then delivered by Dr. C. C. Allen, after which the following was adopted: *Resolved*, That the thanks of this Society be tendered to Dr. Allen for his eloquent and instructive address, and that a committee be appointed to request a copy of it for publication. Said committee consists of Drs. Dodge, Lovejoy and Bridges.

On motion, a committee of three was appointed to take into consideration the expediency of procuring a legal incorporation of this Society, under the act passed at the late session of the legislature. Said committee consists of Allen, Hawes and Bridges.

The following applicants for membership, whose names had been reported by the Recording Secretary, after being vouched for by several members, or recommended by the Executive Committee, were received into membership, viz. A. Hill, of Norwalk, Conn., F. P. Chase, of New York City, and C. H. Stillwell, of Brooklyn, N. Y.

The following preamble and resolution were, on motion, adopted:

*Whereas*, This Society has been informed that certain persons have advertised that they were members of this Society, who were not, therefore, ***Resolved***, That the Secretary be authorised to contradict, in future, all such statements in the same papers in which such advertisements may appear, at the expense of this Society.

The thanks of the Society were presented to Drs. Clark and Lovejoy for their Essays, and to the President for his Address, and copies requested for publication.

The thanks of the Society were also tendered to Dr. Covill, for the able and impartial manner in which he has discharged the duties of President of this Society.

The Society then proceeded to the choice of its officers for the ensuing year, which resulted as follows:

E. Baker, President. C. C. Allen and J. Lovejoy, Vice-presidents. J. G. Ambler, Recording Secretary. T. H. Burras, Corresponding Secretary. George E. Hawes, Treasurer. H. Burdell, Librarian. George Clay, C. D. Brown, Benj. Lord, M. K. Bridges and F. H. Clark, Executive Committee.

Dr. Hill of Norwalk, was appointed to deliver the next annual address, and Drs. Chase and Covill to read Essays before the Society at its next regular meeting. Adjourned sine die.

J. G. AMBLER, *Secretary*.

[*Dental Recorder*.

